# Sleep quality and the integrity of ascending reticular activating system – A multimodal MRI study

**DOI:** 10.1016/j.heliyon.2024.e40192

**Published:** 2024-11-07

**Authors:** Viktória Kokošová, Lubomír Vojtíšek, Marek Baláž, Silvia Mangia, Shalom Michaeli, Pavel Filip

**Affiliations:** a First Department of Neurology, Faculty of Medicine, Masaryk University and University Hospital of St. Anne, Brno, Czech Republic; b Department of Neurology, Faculty of Medicine, Masaryk University and University Hospital Brno, Brno, Czech Republic; c Central European Institute of Technology (CEITEC) Masaryk University, Neuroscience Centre, Brno, Czech Republic; d Center for Magnetic Resonance Research (CMRR), University of Minnesota, Minneapolis, MN, USA; e Department of Neurology, Charles University, First Faculty of Medicine and General University Hospital, Prague, Czech Republic; f Department of Cybernetics, Czech Technical University in Prague, Prague, Czech Republic

**Keywords:** Sleep quality, Brain ageing, Multimodal MRI, Relaxometry, Diffusion weighted imaging

## Abstract

Sleep is crucial for maintaining brain homeostasis and individuals with insufficient sleep are prone to more pronounced brain atrophy as compared to sufficiently sleeping peers. Moreover, sleep quality deteriorates with ageing and ageing is also associated with cerebral structural and functional changes, pointing to their mutual bidirectional interrelationship. This study aimed at determining whether sleep quality and age, separately, affect brain integrity and subsequently, whether sleep significantly modulates the effect of age on brain structural and functional integrity. 113 healthy volunteers underwent a multi-modal MRI imaging to extract information about the microstructure and function of major nodes of the ascending reticular activating system. Sleep quality was assessed by self-administered Pittsburgh's sleep quality index (PSQI) questionnaire. Subject were divided into good (global PSQI score <5) and poor (global PSQI score ≥5) sleep quality group. Whereas only borderline correlations were found between sleep quality and MRI metrics, age exhibited widespread correlations with both functional and microstructural MRI metrics. The latter effect was significantly modulated by sleep quality in ascending reticular activating system, hypothalamus, thalamus and also hippocampus in MRI metrics associated with iron load, cellularity and connectivity, mainly in the subgroup with poor sleep quality. Ergo, our results indicate sleep quality as a substantial contributor to both microstructural and functional brain changes in ageing and call for further research in this emerging topic.

## Highlights

• Sleep quality is a significant contributor to structural and functional changes of brain with ageing in healthy population.
• Numerous microstructural MRI metrics are sensitive to association between sleep quality and ageing in healthy population.
• ARAS, hypothalamus, thalamus and hippocampus were affected by the combined effect of poor sleep quality and ageing.

## Graphical abstract

The presented multimodal MRI study of relationship between sleep, ageing and brain structure shows viable sensitivity of several MRI metrics to sleep quality changes in healthy ageing population in brain areas highly relevant for sleep control as hypothalamus, hippocampus, and major nodes of ascending reticular activating system.

**Figure:**
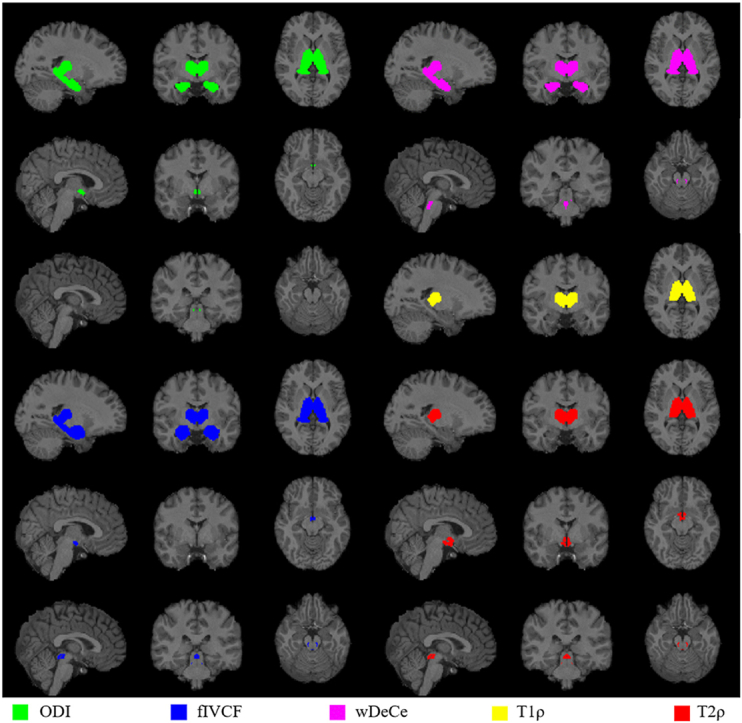

## 1 Introduction

Sleep, the circadian state of reversible unconsciousness, is crucial for maintaining brain homeostasis. During sleep, the brain is cleansed from metabolic waste products and its energy reserves are restored [1,2]. Furthermore, sleep is also suggested to modulate gene expression. Specifically, expression of genes involved in phospholipid synthesis and oligodendrocyte proliferation significantly increases in sleep state, sustaining the integrity of white matter tracts [3]. Moreover, expression of genes regulating antioxidant system [4], immune and stress responses, neuronal plasticity [[5], [6], [7]] and reward-modulatory brain system [8] is sensitive to sleep deprivation. Therefore, the lack of sufficient sleep might lead, via various mechanisms, to the accumulation of toxic substances that potentially trigger chronic inflammatory processes [9], subsequently leading to trophic changes [10,11]. Indeed, individuals with poor sleep quality have been found to suffer from more pronounced brain atrophy compared to people with sufficient sleep [[12], [13], [14], [15]].

The physiology of the sleep-wake cycle depends on structural and functional integrity of numerous brain regions [[16], [17], [18]]. Namely, the system of brain stem and diencephalic nuclei comprises ascending reticular activation system (ARAS) that is active especially in the rapid eye movement (REM) sleep and wakefulness. In addition to ARAS, hypothalamus, thalamus, amygdala and hippocampus are crucial for sleep physiology. The pacemaker of the circadian rhythm - the suprachiasmatic nucleus is located in the hypothalamus together with the ventrolateral preoptic nucleus that is considered as the main sleep initiator, thalamus is the generator of sleep spindles – the hallmark of N2 phase of non-rapid eye movement sleep (NREM) and hippocampus and amygdala, respectively, are suggested to be responsible for sleep-dependent memory consolidation (as reviewed in Ref. [19]). The proper function and structural integrity of these brain regions is critical for the quality of sleep.

A common factor that substantially influences both sleep quality and cerebral structural and functional integrity, is ageing [20]. Nonetheless, the exact nature of how sleep quality and ageing are interrelated with each other is yet to be determined, calling for multimodal studies encompassing both imaging parameters and viable qualitative and/or quantitative descriptors of sleep. Magnetic resonance imaging (MRI) provides such an option, enabling us to detect subtle alterations in both functional and microstructural properties of brain tissue, such as myelin density, cellularity and iron load.

The presented pilot study capitalises on the recent development in high angular resolution diffusion imaging and novel relaxometric protocols, adiabatic longitudinal (T1ρ) and transverse (T2ρ), and non-adiabatic Relaxation Along Fictious Field in the rotating frame of rank 4, RAFF4. Adiabatic T1ρ time constant is suggested to be an indicator of cellular density [21], T2ρ an indicator of iron loads [21,22] and non-adiabatic T_RAFF4_ an indicator of myelin density [23,24]. Further, diffusion weighted imaging (DWI) was used to extract complementary information about tissue microstructure, namely, orientation dispersion index (ODI) and intracellular volume fraction (fICVF) [25]. ODI is considered to be indicator of neurite structural organization, while fICVF is considered to represent the fraction of tissue consisting of axons and dendrites and is suggested to be influenced by microstructural alteration in both neuronal and glial components [25,26]. And lastly, two resting-state functional MRI (rsfMRI) metrics were employed – weighted degree centrality (wDeCe) as a measure of global connectivity [27] and functional resting state fluctuations (fALFF) reflecting the power of spontaneous neural activity [28]. These protocols were utilised to extract functional and microstructural metrics in sleep-relevant deep brain regions: brainstem structures of ascendent arousal network, hypothalamus, and also hippocampus, thalamus and amygdala. Information on sleep quality was acquired from Pittsburgh Sleep Quality Index (PSQI) [29] and based on the score the subjects were divided into good and poor sleep quality group. We did not expect to see any relevant correlation of sleep quality with brain structure and function in the good sleepers. In combination with demographic data, the following main goals were pursued.

1) To determine whether sleep quality and age separately affect MRI metrics as proxies of microstructural and functional integrity of the brain.
2) To evaluate whether sleep quality modulates the relationship between age with both functional and microstructural metrics in sleep-relevant deep brain areas.

We expected sleep quality to decline with increasing age. Furthermore, we hypothesised that low sleep quality would accentuate brain alterations seen with ageing.

## 2 Methods

### 2.1 Subjects

113 healthy volunteers were included in this cross-sectional study. Participants were recruited from the Caucasian population in Czech Republic via the database of Multimodal and functional imaging laboratory of Central European Institute of Technology in Brno from April 2021 to December 2021. Inclusion criteria were age ≥18 years. Exclusion criteria were general contraindications for MRI examination, such as non-MRI compatible metallic implants, claustrophobia and pregnancy. Further, participants with known neurological and/or psychiatric diseases, structural lesion observed on MRI scan and participants with BQRS-M&E ≥ 3 pointing to subjective cognitive end emotional problems were excluded. All participants filled in questionnaire consisted of three main parts: basic demographic information, Brief Questionnaire Regarding Severity of Memory & Emotional Problems (BQRS-M&E) [30], and PSQI questionnaire [29]. All participants signed an informed consent form in accordance with the Declaration of Helsinki and the study was approved by the ethics committee of the University Hospital of St. Anne in Czech Republic. MRI data acquired in this cohort were used for different analyses published in our previous work [31].

### 2.2 Sleep quality assessment

Sleep quality was assessed via PSQI questionnaire, a self-reported, subjective and retrospective measure of sleep quality in the previous four weeks, that evaluates seven domains: sleep latency, sleep duration, subjective sleep quality, sleep efficiency, sleep disturbances, use of sleep medication, and daytime dysfunction. The global PSQI score is calculated as sum of scores of every component [29].

### 2.3 Imaging protocol and data analysis

MRI acquisitions were performed using a 3T Magnetom Prisma system at the Central European Institute of Technology in Brno, Czech Republic. Magnetization-prepared rapid gradient-echo (MP-RAGE) sequence was used for T1-weighted (T1w) acquisitions, with the repetition time (TR) of 2150 ms, time to echo (TE) of 2,47 ms, inversion time (TI) of 1100 ms, voxel size of 1x1x1 mm^3^, flip angle of 8° and generalized autocalibrating partial parallel acquisition (GRAPPA) acceleration factor of 2. For acquisition of T2-weighted images (T2w), SPACE sequence in sagittal orientation, with 1.0 mm isotropic resolution, TR 2.820 ms, TE 72.6 ms and GRAPPA = 2 was utilised. Manufacturer-implemented pre-scan normalized algorithm was employed in both T1w and T2w scans. DWI datasets were acquired using following parameters: TR = 2820 ms, TE = 72.6 ms, multi-band (MB) = 4, in 93 directions, with 7 additional non-diffusion weighted (b0) images, b shells of 750 and 1500 s/mm^2^, voxel size 1.8 x 1.8 × 1.8 mm^3^. The acquisitions were repeated twice, with opposite phase encoding along the antero-posterior and postero-anterior axis. Echo Planar Imaging (EPI) was utilised to obtain rsfMRI data with TR = 900 ms, TE = 30 ms, flip angle 45°, MB = 4. 502 vol in total were acquired using interleaved slice acquisition with 3 mm isotropic resolution and matrix size of 64 x 64. Rotating frame relaxation measurements (adiabatic T1ρ, T2ρ and non-adiabatic RAFF4) were performed using magnetization prepared gradient echo sequence in 30 slices with voxel size of 1.6 x 1.6 × 3.6 mm^3^, TR = 2 s, TE = 3.18 ms, GRAPPA = 3. For adiabatic T1ρ and T2ρ, hyperbolic secant (HS1) pulses were utilised with adiabaticity factor R = 10, pulse duration of 6 ms, bandwidth 1.3 kHz, peak power ꙍ_1_^max^/(2π) = 880 Hz, and number of pulses = 0, 4, 8, 12, 16. For RAFF4 acquisition, the duration of *P*-packet was 4.56 ms, number of *P*-packets in the train 0, 4, 8, 12, 16 and ꙍ_1_^max^/(2π) = 327 Hz [32].

Human Connectome Project (HCP) minimal pre-processing pipeline [33] (was used for structural T1w and T2w scans, DWI and rsfMRI scans pre-processing. The rsfMRI pre-processing HCP minimal pipeline consisted of gradient non-linearity correction, motion correction, B_0_ field in homogeneities correction with the use of spin-echo fieldmaps, rigid-body co-registration to the T1w space with spline interpolation, and 2-mm full width at half maximum (FWHM) surface regularisation and subcortical volume smoothing. Further, rsfMRI processing utilised HCP rsfMRI pipeline [34] including MELODIC independent component analysis and automatic artefactual components identification via the FIX algorithm, motion-related time-courses were regressed out. HCP training data were utilised as the baseline for the automatic classification algorithm with output manual correction and regression of the artefactual components’ contribution from the rsfMRI data. The AFNI package [35] was utilised for the calculation of voxel-wise wDeCe with sparsity threshold of 0.1 and fALFF as the total power within the frequency range between 0.001 and 0.1 Hz [28]. For DWI processing the HCP pipeline included the optional gradient non-linearity correction and the HCP-pipeline output was utilised for NODDI parameter calculation [36]. The image processing of adiabatic T1ρ, T2ρ and RAFF4 employed 2-step 3D-rigid body motion correction with co-registration of all acquired scans to the first acquisition of the relevant sequence (as implemented in the FSL 6.0 MCFLIRT), relaxation time constant calculation with 2-parameter non-linear fitting for T1ρ and T2ρ scans, and 4-parameter non-linear fitting for RAFF4, followed by rigid body registration to the T1w scan [32].

**Fig. 1:**
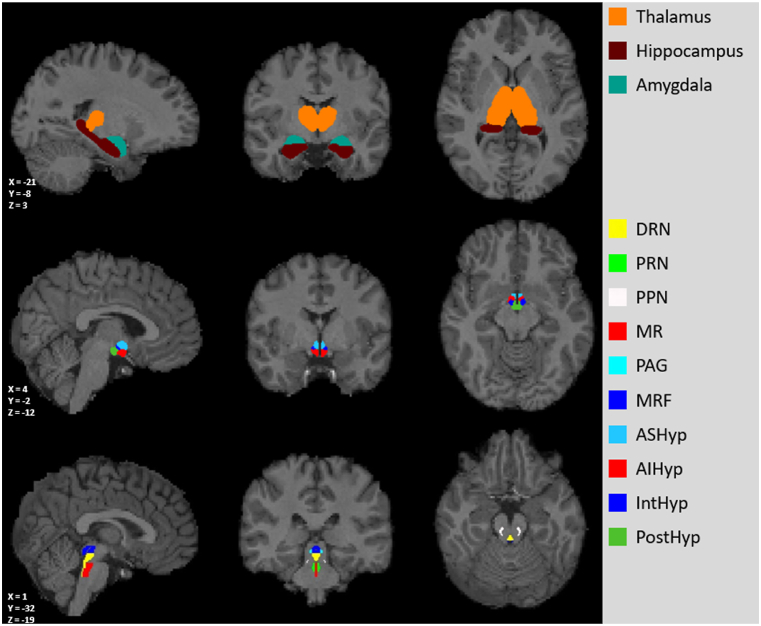
Regions of interest visualised from one representative subject in sagittal, coronal and transverse plane. Slice coordinates provided in left bottom corners. DRN – the dorsal raphe nucleus, PRN – the rostral pontine reticular nucleus, PPN – pedunculopontine tegmental nucleus, MR – the median raphe nucleus, PAG – periaqueductal grey matter, MRF – mesencephalic reticular formation, PRN – the rostral pontine reticular neucleus, ASHyp – anterior superior hypothalamus, AIHyp – anterior inferior hypothalamus, IntHyp – intermediate hypothalamus, PostHyp – posterior hypothalamus.

Ascending Arousal Network Atlas (Martinos Center for Biomedical Imaging, Charlestown, MA; https://www.nmr.mgh.harvard.edu/resources/aan-atlas) (Edlow et al., 2012) (AAN) was used to define 6 regions of interest (ROIs) (Fig. 1): periaqueductal grey matter (PAG), pedunculopontine tegmental nucleus (PPN), the dorsal raphe nucleus (DRN), mesencephalic reticular formation (MRF), the median raphe nucleus (MR), the rostral pontine reticular nucleus (PRN). The hypothalamus subsegmentation (4 ROIs) into anterior superior hypothalamus (ASHyp), anterior inferior hypothalamus (AIHyp), intermediate hypothalamus (IntHyp) and posterior hypothalamus (PostHyp) was obtained using Hypothalamus Atlas (https://github.com/SpindM/HypothalamicAtlas) [37]. Furthermore, FreeSurfer-generated masks of thalamus, amygdala and hippocampus (3 ROIs) were utilised.

The accuracy and quality of processing and segmentation outputs were evaluated by visual inspection of two trained operators (V.K. and P.F.). Further, to exclude subjects with framewise motion surpassing the range of two voxels and/or anatomical variation, the root-mean-squared voxel displacement evaluation for motion correction in DWI, rsfMRI, T1ρ, T2ρ and RAFF4 maps was utilised.

### 2.4 Statistical analysis

All statistical analyses were carried out by using Matlab software v. R2022a. Based on global PSQI scores, the subjects were binarized into two groups – normal (global PSQI <5) and poor sleep quality (global PSQI score ≥5) group [29]. Spearman's correlation coefficient was utilised to test for correlation between age and Global PSQI in the whole cohort and in binarized groups.

Medians of individual MRI metrics (T1ρ, T2ρ, RAFF4, ODI, fICVF, wDeCe and fALFF) were calculated over each of the 13 ROIs in each subject. As age and sleep quality are considered to be interdependent, to test the hypotheses, three separate general linear models (GLMs) were created and permutation-based non-parametric analysis as implemented in the Permutation Analysis of Linear Models package [38] with use of permutation-based Pearson correlation coefficient as the test statistics was utilised. For Aim 1, two general linear models (GLM) were created with medians of individual MRI metrics over individual ROIs as a dependent variables, sex as covariate of non-interest, and age as independent variable in first GLM and global PSQI in the second GLM. To analyse the effect of interaction between age and global PSQI (Aim 2) third GLM was built with medians of MRI metrics over individual ROIs as dependent variables, interaction between age and global PSQI as independent variables and sex as covariates of non-interest. Age was treated as a continuous variable in all analyses. For multiple testing correction we implemented False discovery rate (FDR) correction for each of the two aims separately, with alpha of 0.05 as the type I error threshold.

## 3 Results

Nine participants were excluded because of missing and/or incomplete questionnaire data. Based on subjective cognitive problems according to BQRS-M&E score, we excluded 2 more participants. And finally, due to MRI data errors further 6 subjects (2 for excessive motion and 4 for incomplete acquisitions) were excluded, leaving in total 96 subjects for the final analysis.

Basic demographic data are summarised in Table 1. Global PSQI score showed a trend to increase with advanced age (Fig. 1 in supplementary material), however the correlation was not significant either in the whole group (rho = 0.019; p > 0.20), nor in groups after binarization (rho = −0.036 and 0.151 for normal sleep quality group and poor sleep quality group, respectively; p > 0.20 in both subgroups). This absence of significant correlation is likely due to a low number of subjects with global PSQI greater than 8.

**Table 1:** Summary of basic demographic data (total N, age in years and global PSQI score) for the whole group and groups after binarization – normal (global PSQI <5) and poor sleep quality group (global PSQI ≥5). The values are stated in the format median (range).

Whole group		Males	Females
Total N	96	48	48
Age	44 (19–89)	45.5 (19–89)	42 (20–75)
Global PSQI	4 (0–14)	4 (1–12)	4 (0–14)
**Normal sleep quality group**			
Total N	53	25	28
Age	44.2 (20–75)	44 (24–74)	41 (20–75)
Global PSQI	3 (0–4)	3 (1–4)	3 (1–4)
**Poor sleep quality group**			
Total N	43	23	20
Age	46 (19–89)	47 (19–89)	43.5 (20–74)
Global PSQI	6 (5–14)	6 (5–12)	6.5 (5–14)

### 3.1 Correlations of sleep quality and age with MRI metrics

Age exhibited widespread correlations with MRI metrics (Table 2). In both subgroups (normal and poor sleep quality), hypothalamus, thalamus and hippocampus exhibited significant age-related differences. However, in the subgroup with normal sleep quality, there were no or only borderline age effects in AAN ROIs (T2ρ in PPN and MRF, fICVF in PAG) and no significant correlations between age and functional MRI metrics in any of the studied ROIs. As for the association between the sleep quality and MRI metrics, there were only minimal findings (hippocampus ODI and wDeCe and T1ρ in PAG in the group of subjects with normal sleep quality; fICVF in PAG in the group of poor sleep quality subjects) (Table 3).

**Table 2:** Association between age and individual MRI metrics, separately for the subgroups with normal (A) and poor (B) sleep quality. Pearson correlation coefficients and FDR corrected p-values are stated in form r (p-value). Bold type indicates statistically significant results (p < 0.05). ODI – orientation dispersion index, fICVF – intracellular volume fraction, fALFF – functional amplitude of resting state fluctuations, wDeCe – weighted degree centrality, PAG - periaqueductal grey matter, PPN - pedunculopontine tegmental nucleus, DRN - the dorsal raphe nucleus, MR - the median raphe nucleus, MRF - mesencephalic reticular formation, PRN - the rostral pontine reticular nucleus, ASHy - anterior superior hypothalamus, AIHyp - anterior inferior hypothalamus, IntHyp - intermediate hypothalamus, PostHyp - posterior hypothalamus.

A	M1	T1ρ	T2ρ	RAFF4	ODI	fICVF	fALFF	wDeCe
Global PSQI <5	PAG	0.28 (0.04)	0.20 (0.15)	−0.01 (0.94)	0.07 (0.62)	**0.27 (0.04)**	−0.06 (0.66)	−0.05 (0.74)
	PPN	0.15 (0.27)	**0.30 (0.03)**	0.06 (0.73)	0.24 (0.07)	0.16 (0.22)	−0.13 (0.34)	−0.17 (0.24)
	DRN	0.22 (0.12)	0.22 (0.11)	−0.19 (0.17)	−0.13 (0.32)	0.20 (0.15)	−0.15 (0.30)	−0.19 (0.19)
	MR	0.13 (0.40)	0.19 (0.19)	0.20 (0.16)	0.06 (0.66)	0.20 (0.15)	−0.10 (0.46)	0.05 (0.69)
	MRF	0.26 (0.05)	**0.27 (0.04)**	0.16 (0.23)	0.08 (0.58)	0.04 (0.77)	−0.01 (0.96)	0.20 (0.15)
	PRN	0.05 (0.73)	0.22 (0.08)	0.18 (0.17)	−0.23 (0.10)	−0.08 (0.57)	−0.14 (0.30)	0.00 (0.97)
	AIHyp	0.24 (0.07)	**0.30 (0.02)**	0.12 (0.39)	**−0.34 (0.01)**	**0.36 (0.01)**	0.14 (0.31)	0.01 (0.96)
	ASHyp	**0.55 (1.0E-04)**	**0.50 (3.0E-04)**	**0.59 (1.0E-04)**	**0.37 (0.01)**	**0.38 (0.01)**	0.04 (0.74)	0.05 (0.74)
	IntHyp	0.06 (0.68)	0.20 (0.13)	0.01 (0.96)	−0.17 (0.20)	0.12 (0.39)	0.19 (0.17)	0.03 (0.80)
	Phyp	**0.33 (0.01)**	**0.39 (3.8E-03)**	0.26 (0.06)	−0.03 (086)	**0.27 (0.04)**	0.04 (0.76)	−0.13 (0.35)
	Thalamus	**0.32 (0.02)**	**0.59 (1.0E-04)**	−0.02 (0.87)	**−0.55 (2.0E-04)**	0.26 (0.06)	0.02 (0.89)	0.22 (0.11)
	Hippocampus	0.22 (0.11)	**0.46 (4.0E-04)**	0.18 (0.19)	**0.44 (8.0E-04)**	0.03 (0.85)	−0.12 (0.41)	−0.01 (0.96)
	Amygdala	0.13 (0.36)	**0.36 (0.01)**	−0.11 (0.44)	−0.03 (0.86)	−0.04 (0.79)	0.01 (0.92)	0.13 (0.36)

B	M1	T1ρ	T2ρ	RAFF4	ODI	fICVF	fALFF	wDeCe
Global PSQI ≥5	PAG	0.20 (0.20)	**0.45 (1.8E-03)**	0.22 (0.14)	0.01 (0.96)	**0.36 (0.01)**	0.14 (0.37)	0.21 (0.17)
	PPN	0.23 (0.11)	**0.52 (3.0E-04)**	−0.08 (0.60)	−0.21 (0.17)	0.26 (0.07)	0.29 (0.05)	**0.32 (0.02)**
	DRN	0.19 (0.22)	0.07 (0.66)	0.11 (0.47)	−0.29 (0.06)	0.21 (0.16)	0.25 (0.10)	**0.28 (0.03)**
	MR	0.12 (0.47)	0.14 (0.40)	0.05 (0.73)	0.20 (0.20)	0.25 (0.11)	0.14 (0.36)	**0.38 (4.5E-03)**
	MRF	**0.37 (0.02)**	**0.59 (1.0E-04)**	0.22 (0.16)	**0.50 (2.0E-04)**	−0.18 (0.25)	0.19 (0.23)	0.12 (0.47)
	PRN	−0.09 (0.57)	0.13 (0.42)	−0.33 (0.03)	−0.02 (0.92)	**0.35 (0.02)**	0.18 (0.24)	**0.50 (5.0E-04)**
	AIHyp	**0.52 (4.0E-04)**	**0.55 (4.0E-04)**	0.09 (0.54)	−0.21 (0.17)	0.28 (0.05)	**0.34 (0.02)**	**0.30 (0.04)**
	ASHyp	**0.48 (6.0E-04)**	**0.49 (4.0E-04)**	**0.40 (3.7E-03)**	**0.55 (3.0E-04)**	0.25 (0.11)	0.21 (0.17)	0.14 (0.39)
	IntHyp	0.11 (0.47)	0.07 (0.67)	0.02 (0.88)	−0.16 (0.30)	0.05 (0.73)	0.27 (0.07)	0.30 (0.05)
	Phyp	**0.56 (2.0E-04)**	**0.61 (1.0E-04)**	**0.50 (3.0E-04)**	−0.07 (0.63)	**0.40 (0.01)**	0.24 (0.11)	0.24 (0.08)
	Thalamus	**0.48 (8.0E-04)**	**0.69 (1.0E-04)**	0.02 (0.90)	**−0.53 (3.0E-04)**	**0.37 (0.01)**	−0.05 (0.73)	**0.43 (3.6E-03)**
	Hippocampus	0.20 (0.21)	**0.43 (3.3E-03)**	−0.08 (0.63)	**0.54 (1.0E-04)**	**0.51 (6.0E-04)**	0.08 (0.63)	**0.52 (2.0E-04)**
	Amygdala	0.05 (0.77)	0.30 (0.05)	−0.20 (0.19)	−0.08 (0.61)	**0.52 (9.0E-04)**	0.15 (0.35)	−0.05 (0.81)

**Table 3:** Association between sleep quality (global PSQI) and individual MRI metrics, separately for the subgroups with normal (A) and poor (B) sleep quality. Pearson correlation coefficients and FDR corrected p-values are stated in form r (p-value. Bold type indicates statistically significant results (p < 0.05). ODI – orientation dispersion index, fICVF – intracellular volume fraction, fALFF – functional amplitude of resting state fluctuations, wDeCe – weighted degree centrality, PAG - periaqueductal grey matter, PPN - pedunculopontine tegmental nucleus, DRN - the dorsal raphe nucleus, MR - the median raphe nucleus, MRF - mesencephalic reticular formation, PRN - the rostral pontine reticular nucleus, ASHy - anterior superior hypothalamus, AIHyp - anterior inferior hypothalamus, IntHyp - intermediate hypothalamus, PostHyp - posterior hypothalamus.

A	M2	T1ρ	T2ρ	RAFF4	ODI	fICVF	fALFF	wDeCe
Global PSQI <5	PAG	**−0.28 (0.04)**	−0.05 (0.72)	−0.16 (0.25)	0.20 (0.16)	−0.12 (0.36)	0.03 (0.84)	0.19 (0.16)
	PPN	−0.07 (0.59)	−0.24 (0.08)	−0.17 (0.20)	−0.02 (0.87)	−0.02 (0.89)	0.17 (0.20)	0.13 (0.35)
	DRN	0.02 (0.89)	0.03 (0.83)	0.07 (0.63)	0.10 (0.46)	0.15 (0.26)	0.03 (0.85)	0.13 (0.38)
	MR	0.02 (0.88)	−0.07 (0.65)	−0.02 (0.90)	−0.17 (0.23)	−0.14 (0.32)	0.10 (0.46)	0.12 (0.40)
	MRF	−0.10 (0.47)	0.02 (0.87)	0.03 (0.83)	0.20 (0.16)	0.07 (0.64)	0.02 (0.87)	0.17 (0.22)
	PRN	−0.10 (0.44)	0.01 (0.94)	0.11 (0.40)	−0.06 (0.66)	−0.01 (0.94)	0.09 (0.53)	0.14 (0.30)
	AIHyp	0.01 (0.93)	0.02 (0.89)	0.13 (0.39)	0.09 (0.51)	0.06 (0.69)	−0.01 (0.92)	0.15 (0.24)
	ASHyp	0.01 (0.97)	−0.09 (0.54)	−0.09 (0.54)	0.16 (0.25)	0.12 (0.37)	−0.09 (0.49)	0.04 (0.75)
	IntHyp	−0.12 (0.40)	−0.18 (0.18)	0.01 (0.94)	−0.08 (0.57)	0.01 (0.94)	0.11 (0.43)	0.11 (0.42)
	Phyp	−0.24 (0.08)	−0.06 (0.68)	−0.03 (0.81)	−0.03 (0.85)	0.03 (0.82)	0.04 (0.74)	0.20 (0.13)
	Thalamus	−0.04 (0.75)	−0.05 (0.75)	0.01 (0.92)	0.14 (0.30)	0.09 (0.54)	0.08 (0.54)	0.17 (0.20)
	Hippocampus	−0.04 (0.78)	−0.06 (0.67)	−0.01 (0.91)	**−0.32 (0.02)**	−0.05 (0.74)	0.17 (0.21)	**0.32 (0.02)**
	Amygdala	−0.11 (0.42)	−0.15 (0.28)	0.05 (0.75)	0.25 (0.07)	0.12 (0.40)	0.14 (0.30)	0.19 (0.15)

B	M2	T1ρ	T2ρ	RAFF4	ODI	fICVF	fALFF	wDeCe
Global PSQI ≥5	PAG	−0.10 (0.50)	0.00 (0.98)	−0.06 (0.71)	−0.10 (0.51)	**0.32 (0.02)**	−0.04 (0.78)	0.02 (0.89)
	PPN	0.09 (0.55)	0.05 (0.76)	−0.09 (0.58)	0.02 (0.90)	0.25 (0.09)	−0.04 (0.78)	0.14 (0.25)
	DRN	−0.12 (0.46)	0.00 (0.98)	−0.19 (0.22)	−0.01 (0.96)	0.19 (0.21)	−0.04 (0.78)	0.10 (0.49)
	MR	−0.05 (0.70)	−0.07 (0.60)	−0.05 (0.75)	−0.13 (0.38)	0.01 (0.95)	−0.07 (0.65)	0.19 (0.14)
	MRF	0.07 (0.67)	−0.11 (0.50)	−0.09 (0.58)	0.01 (0.97)	0.10 (0.49)	−0.06 (0.68)	−0.08 (0.56)
	PRN	−0.01 (0.97)	0.11 (0.48)	−0.10 (0.53)	0.00 (1.00)	0.15 (0.30)	−0.02 (0.88)	0.27 (0.06)
	AIHyp	−0.06 (0.71)	0.05 (0.74)	−0.20 (0.19)	0.30 (0.05)	0.04 (079)	−0.15 (0.33)	−0.03 (0.87)
	ASHyp	−0.04 (0.79)	0.00 (0.98)	−0.03 (0.83)	0.13 (0.42)	0.16 (0.33)	−0.20 (0.19)	0.01 (0.91)
	IntHyp	0.07 (0.65)	−0.20 (0.19)	−0.27 (0.08)	−0.17 (0.26)	0.07 (0.64)	−0.16 (0.29)	0.02 (0.87)
	Phyp	−0.12 (0.46)	0.02 (0.92)	−0.10 (0.51)	0.07 (0.67)	0.26 (0.09)	−0.10 (0.49)	0.05 (0.73)
	Thalamus	0.09 (0.58)	0.00 (0.99)	−0.09 (0.56)	−0.10 (0.51)	0.20 (0.17)	−0.21 (0.17)	0.16 (0.30)
	Hippocampus	0.00 (0.98)	−0.04 (0.79)	−0.15 (0.34)	0.18 (0.25)	0.19 (0.20)	−0.19 (0.22)	0.22 (0.10)
	Amygdala	−0.11 (0.49)	−0.13 (0.40)	−0.27 (0.08)	0.04 (0.82)	0.17 (0.28)	−0.29 (0.06)	0.02 (0.92)

### 3.2 Effect of interaction between sleep quality and ageing on MRI metrics

**Fig. 2:**
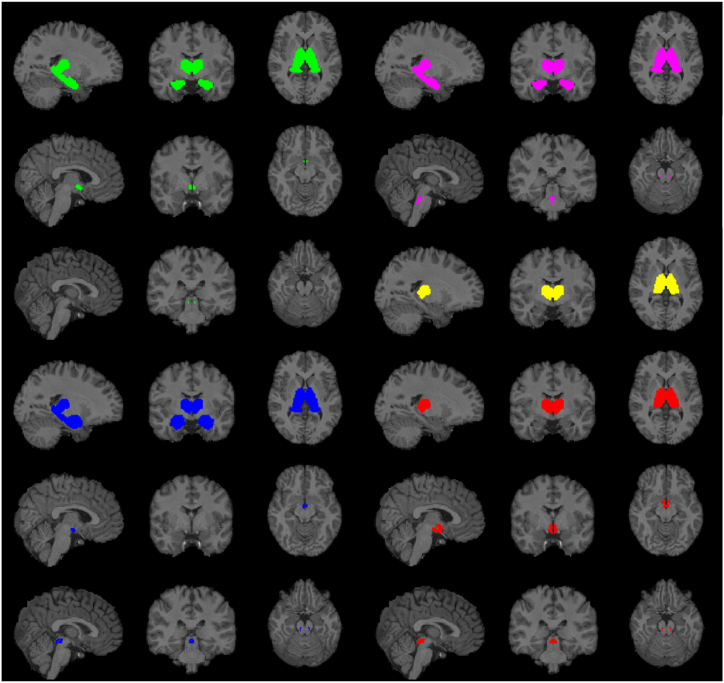
Visualisation of regions of interest with significant correlation between age and global PSQI with MRI metrics: ODI (green), fIVCF (blue), wDeCe (pink), T1ρ (yellow) and T2ρ (red) in poor sleep quality group. ODI – orientation dispersion index, fICVF – intracellular volume fraction, wDeCe – weighted degree centrality. (For interpretation of the references to colour in this figure legend, the reader is referred to the Web version of this article.)

In the group with normal sleep quality, there was a significant age and global PSQI interaction in hypothalamus in several microstructural MRI metrics (T1ρ, T2ρ and ODI in ASHyp, fICVF in AIHyp), but functional MRI metrics failed to yield any significant results in either of ROIs (Table 4A). On the other hand, in the group of subjects with poor sleep quality, there were significant interactions in AAN ROIs, hypothalamus, thalamus, hippocampus and also amygdala (Table 4B). Multiple MRI modalities provided statistically significant results, mostly T2ρ, fICVF, ODI and wDeCe (Fig. 2).

**Table 4:** Interaction of sleep quality (global PSQI) and age vs individual MRI metrics, separately for the subgroups with normal (A) and poor (B) sleep quality. Pearson correlation coefficients and FDR corrected p-values are stated in form r (p-value). Bold type indicates statistically significant results. ODI – orientation dispersion index, fICVF – intracellular volume fraction, fALFF – functional amplitude of resting state fluctuations, wDeCe – weighted degree centrality, PAG - periaqueductal grey matter, PPN - pedunculopontine tegmental nucleus, DRN - the dorsal raphe nucleus, MR - the median raphe nucleus, MRF - mesencephalic reticular formation, PRN - the rostral pontine reticular nucleus, ASHy - anterior superior hypothalamus, AIHyp - anterior inferior hypothalamus, IntHyp - intermediate hypothalamus, PostHyp - posterior hypothalamus.

A	M3	T1ρ	T2ρ	RAFF4	ODI	fICVF	fALFF	wDeCe
Global PSQI <5	PAG	−0.05 (0.70)	0.10 (0.49)	−0.11 (0.43)	0.24 (0.09)	0.11 (0.40)	−0.05 (0.70)	0.08 (0.57)
	PPN	0.01 (0.97)	0.03 (0.83)	−0.09 (0.56)	0.18 (0.18)	0.13 (0.33)	0.01 (0.96)	−0.06 (0.68)
	DRN	0.11 (0.46)	0.16 (0.25)	−0.07 (0.62)	0.02 (0.90)	0.27 (0.05)	−0.13 (0.36)	−0.08 (0.62)
	MR	0.09 (0.54)	0.04 (0.79)	0.08 (0.56)	−0.13 (0.34)	0.02 (0.87)	0.00 (0.99)	0.10 (0.47)
	MRF	0.07 (0.60)	0.19 (0.17)	0.11 (0.43)	0.26 (0.06)	0.10 (0.49)	−0.04 (0.79)	0.23 (0.10)
	PRN	−0.08 (0.55)	0.11 (0.37)	0.17 (0.19)	−0.21 (0.12)	−0.03 (0.84)	−0.05 (0.70)	0.08 (0.57)
	AIHyp	0.20 (0.14)	0.25 (0.05)	0.20 (0.15)	−0.19 (0.18)	**0.31 (0.02)**	0.03 (0.83)	0.11 (0.45)
	ASHyp	**0.35 (0.01)**	0.24 (0.09)	**0.29 (0.04)**	**0.39 (3.3E-03)**	0.35 (0.35)	−0.10 (0.46)	0.00 (0.99)
	IntHyp	−0.05 (0.72)	0.01 (0.96)	0.01 (0.93)	−0.17 (0.21)	0.07 (0.65)	0.18 (0.19)	0.07 (0.64)
	Phyp	−0.01 (0.92)	0.19 (0.17)	0.15 (0.29)	−0.05 (0.72)	0.21 (0.12)	0.01 (0.97)	0.02 (0.91)
	Thalamus	0.12 (0.38)	**0.35 (0.01)**	−0.07 (0.59)	−0.25 (0.08)	0.29 (0.04)	0.09 (0.53)	0.26 (0.06)
	Hippocampus	0.09 (0.53)	0.26 (0.06)	0.08 (0.56)	0.05 (0.73)	−0.02 (0.90)	0.05 (0.72)	0.21 (0.13)
	Amygdala	−0.01 (0.94)	0.12 (0.38)	−0.07 (0.62)	0.20 (0.14)	0.07 (0.63)	0.10 (0.47)	0.20 (0.14)

B	M3	T1ρ	T2ρ	RAFF4	ODI	fICVF	fALFF	wDeCe
Global PSQI ≥5	PAG	0.07 (0.63)	**0.33 (0.03)**	0.10 (0.49)	−0.02 (0.91)	**0.44 (1.9E-03)**	0.09 (0.56)	0.16 (0.24)
	PPN	0.19 (0.16)	**0.37 (0.01)**	−0.10 (0.51)	−0.14 (0.35)	**0.36 (0.02)**	0.18 (0.22)	**0.31 (0.04)**
	DRN	0.05 (0.73)	0.01 (0.93)	−0.05 (0.76)	−0.19 (0.21)	0.27 (0.08)	0.14 (0.33)	0.25 (0.07)
	MR	0.07 (0.67)	0.06 (0.72)	0.03 (0.85)	0.06 (0.70)	0.17 (0.22)	0.01 (0.93)	**0.37 (0.03)**
	MRF	0.29 (0.07)	**0.34 (0.03)**	0.11 (0.50)	**0.36 (0.01)**	−0.07 (0.64)	0.09 (0.55)	0.05 (0.75)
	PRN	−0.05 (0.73)	0.16 (0.29)	−0.25 (0.10)	−0.01 (0.94)	0.28 (0.06)	0.07 (0.66)	**0.50 (1.0E-03)**
	AIHyp	0.29 (0.05)	**0.38 (0.01)**	−0.03 (0.83)	0.07 (0.64)	0.22 (0.13)	0.13 (0.42)	0.16 (0.25)
	ASHyp	0.31 (0.05)	**0.34 (0.03)**	0.28 (0.06)	**0.45 (2.3E-03)**	0.22 (0.22)	0.02 (0.89)	0.09 (0.57)
	IntHyp	0.13 (0.42)	−0.05 (0.75)	−0.15 (0.32)	−0.20 (0.18)	0.09 (0.56)	0.09 (0.55)	0.20 (0.16)
	Phyp	0.28 (0.07)	**0.39 (0.01)**	0.28 (0.06)	0.03 (0.84)	**0.41 (0.01)**	0.10 (0.51)	0.20 (0.12)
	Thalamus	**0.39 (0.01)**	**0.48 (1.6E-03)**	0.01 (0.94)	**−0.43 (4.4E-03)**	**0.32 (0.02)**	−0.19 (0.23)	**0.39 (0.01)**
	Hippocampus	0.17 (0.28)	0.28 (0.07)	−0.10 (0.54)	**0.46 (1.9E-03)**	**0.46 (9.0E-04)**	−0.09 (0.55)	**0.51 (0.01)**
	Amygdala	1.0E-03 (0.99)	0.15 (0.33)	−0.23 (0.13)	−0.02 (0.90)	**0.42 (4.6E-03)**	−0.12 (0.45)	−0.04 (0.80)

## 4 Discussion

The main goal of our investigation was to study the relationship between sleep quality and ageing in regard to structural and functional changes of ARAS. To the best of our knowledge, this is the first study employing complex multimodal MRI protocol with metrics sensitive to iron, myelination, neural density and function in the study of relationship between sleep quality and ageing in ARAS.

The widespread correlation with age is generally an expectable finding in all the employed MRI metrics [31]. Interestingly, the correlations between MRI metrics and age in ARAS ROIs were far more common and pronounced in the subgroup with poor sleep quality than in the subgroup with normal sleep quality. This finding was corroborated also in the analysis of the interaction of age and sleep quality, where substantial affections were detected in ARAS, hypothalamus, thalamus and also hippocampus mostly in the subgroup with global PSQI ≥5, while virtually only hypothalamus yielded significant results in the subgroup with normal sleep quality (see Table 4). Several MRI metrics emerged as uniquely sensitive to this association, namely T2ρ, fICVF, ODI and wDeCe. Unexpectedly, there was no significant association between subjective sleep quality and ageing in our study. Lack of subjects with very poor subjective sleep quality in our cohort may be one of possible contributors to this finding.

The presented study shows a rather consistent association of T2ρ, as an indicator of iron load [21,22,39], with interaction between sleep quality and ageing in multiple ARAS and hypothalamic subregions, only in the group with poor sleep quality. T2ρ has been shown to be highly sensitive not only to the microstructural changes seen in ageing [31], but also in various pathologies [40,41]. Since iron is an important factor in multiple essential processes, ranging from oxygen transportation to neurotransmitter metabolism [42], the association of age, poor sleep quality and abnormal iron homeostasis presented here has far-reaching implications. Further studies will be highly desirable to evaluate potential link of this finding to the risk of the full development of neurodegenerative conditions in poor sleepers.

fICVF has emerged significant in the analysis of the interaction of age and sleep quality, but only in the poor sleep quality subgroup, in thalamus, hippocampus and amygdala. This observation points to presence of microstructural changes on the level of axons, dendrites, but also neuronal and glial components, that are more profound in poor sleepers with ageing. These changes are not unique only for association between sleep quality and ageing and increased fICVF was observed in numerous pathological conditions, such as mild traumatic brain injury [43], Alzheimer's disease [44] and inflammatory states [45] with structural reorganization [43], decrease in grey matter volume with subsequent increase in its density [44] and cellular swelling [45]suggested as possible biological underpinnings of fICVF increase.

ODI, as a spatial indicator of neurite structural organization, showed a complementary finding, namely a significant correlation between the interaction of age and sleep quality and ODI in the subgroup with poor sleep quality again in thalamus and hippocampus. This finding corroborates the above mentioned microstructural changes mirrored by fICVF changes, however the evaluation of the biological underpinning of this effect is rather complicated. It was suggested, that increase in ODI in subcortical areas in process of ageing could mirror compensatory mechanisms in endeavour to preserve its function [46,47]. However, axonal disorganization could drive the ODI increase as well [[48], [49], [50]].

T1ρ, which is suggested to mirror the cellular density [21,51], showed significant correlation with the interaction of age and sleep quality only in thalamus in the poor sleep quality group. This result is consistent with aforementioned correlations between the age and sleep quality interaction with fICVF and ODI, another markers of tissue microstructure, in thalamus. Altogether, those findings point to a high vulnerability of thalamus to microstructure changes due to sleep quality.

In the poor sleep quality group we did not observe a significant correlation between sleep quality or its interaction with age with RAFF4, a marker of myelin density [23] and integrity [52]. Genes promoting oligodendrocytes precursor cells proliferation, myelination and phospholipid synthesis are preferentially transcribed during sleep [3], implying the role of sleep in sustaining the myelin integrity. However, in a study of chronic stress in animal model, there was significant change in oligodendrocyte morphology, e.g. reduction of the nodal and paranodal lengths, but preserved myelin thickness [53]; it is possible that a similar mechanism is implied in poor sleep quality subjects thus explaining the lack of RAFF4 findings known to be sensitive to both myelin content and integrity [54].

wDeCe, as a proxy of global brain connectivity, provided further interesting insights. In models where we tested the correlations of age alone and the correlations of its interaction with sleep quality with structural and functional changes, we observed significant results on functional MRI metrics only in the group of poor sleep quality subjects, and significant correlations were always positive, indicating increase in functional connectivity in poor sleep quality group. This phenomenon of increased functional connectivity in subcortical areas was observed also in patients with obstructive sleep apnea [55] and possibly the aforementioned compensatory mechanisms are accountable for these observations.

Lastly, limitations to this study must be considered as well. The inference of underlying biological processes leading to the changes in detected MRI signal are complicated at the best, and require caution in their interpretation. MRI is generally considered a very sensitive, but rather poorly specific tool. This may be seen in the analysis of the separate effect of global PSQI, revealing unexpectedly several associations mainly in the subgroup with normal sleep quality, which were not mirrored in the subgroup with poor sleep quality. This rather counter-intuitive result must be seen in the light of subjective nature of the PSQI scale, the low number of subjects with severe sleep quality deterioration in the current study, and also generally borderline statistical significances of the findings. While studies employing several MRI modalities provide multifaceted results with partly overlapping sensitivity to underlying biology, they reveal also the caveats of single-modality studies basing their outcomes on one MRI parameter. On the other hand, this repeated emergence of several findings in multiple modalities and/or ROIs provides much stronger vindication of the claim. Secondly, ARAS consists of several nuclei, some of them of relatively small size, therefore only nuclei providing reasonable signal coverage could be included, that precluded areas such as locus coeruleus from the analysis. However, currently there is effort to improve the resolution of adiabatic relaxation metrics [56]. Further, in our analyses we took into account sex as a cofounding factor, however other possible cofounds such as smoking, alcohol intake, exercise, household and economic conditions should be taken under consideration in future studies with larger cohorts of participants. Last but not least, the pilot nature of our data based on subjective and retrospective assessment of sleep quality using a sleep questionnaire definitely calls for further confirmation in preferably longitudinal studies in larger cohorts of subjects with objective measurements of sleep quality, e.g. actigraphy.

## 5 Conclusions

Our study indicates sleep quality as a significant contributor to both structural and functional brain changes in healthy ageing population. Alterations in iron load, cellularity and function of several structures crucial for sleep have been implicated with age mainly in the population with subjective poor sleep quality. To disentangle the causality of relationship between brain changes and sleep quality in ageing longitudinal studies with preferably objective measurements of sleep quality, e.g. actigraphy, are required.

## CRediT authorship contribution statement

**Viktória Kokošová:** Writing – original draft, Visualization, Validation, Resources, Methodology, Funding acquisition, Formal analysis, Data curation, Conceptualization. **Lubomír Vojtíšek:** Writing – review & editing, Resources, Project administration, Methodology, Investigation, Data curation, Conceptualization. **Marek Baláž:** Writing – review & editing. **Silvia Mangia:** Writing – review & editing, Methodology, Funding acquisition, Conceptualization. **Shalom Michaeli:** Writing – review & editing, Methodology, Funding acquisition, Conceptualization. **Pavel Filip:** Writing – review & editing, Validation, Supervision, Methodology, Funding acquisition, Formal analysis, Data curation, Conceptualization.

## Data availability

Raw or processed data of this study are not publicly available due to the sensitive nature of human data acquired in subjects. The data is available upon reasonable request to the corresponding author.

## Ethics approval

The ethics committee of the University Hospital of St. Anne, Brno, Czech Republic approved the study protocol.

## Informed consent to participate

Each participant provided written informed consent in accordance with the Declaration of Helsinki.

## Declaration of competing interest

The authors declare that they have no known competing financial interests or personal relationships that could have appeared to influence the work reported in this paper.
