# Supplementary material for: Sleep quality and the integrity of ascending reticular activating system – A multimodal MRI study
Source: Heliyon. 2024 Nov 7;10(22):e40192. doi: 10.1016/j.heliyon.2024.e40192 (PMC11693918; doi:10.1016/j.heliyon.2024.e40192)
Supplement: Multimedia component 1 [file mmc1.docx]

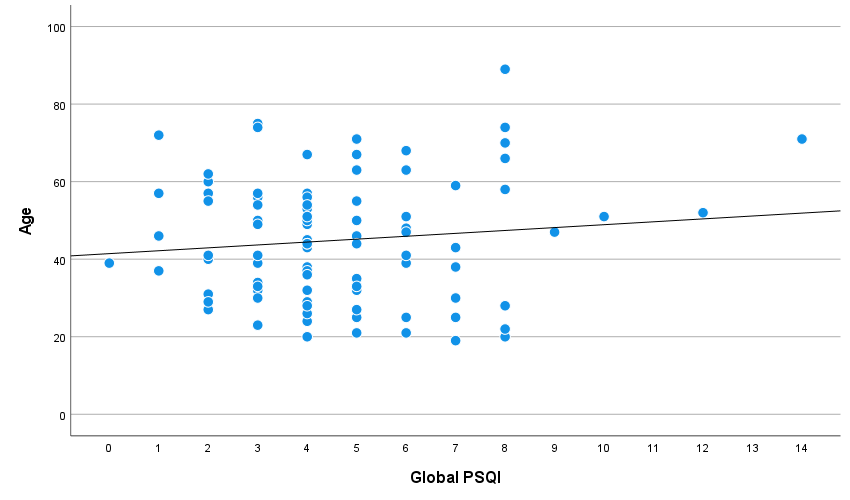


**Figure 1:** 2D scatter plot with overlaid linear trendline for correlation between age (Y axis) and global PSQI (X axis) in the group of 96 subjects with complete MRI dataset.

| **Whole group** | **T1ρ [ms]** | **T2ρ [ms]** | **RAFF4 [ms]** | **ODI** | **fICVF** | **fALFF** | **wDeCe** |
| --- | --- | --- | --- | --- | --- | --- | --- |
| PAG | 191.404 ±11.5 | 103.007 ±10.6 | 436.133  ±28.6 | 0.274  ±0.1 | 0.437  ±0.1 | 0.412  ±0.1 | 57.074 ±186.9 |
| PPN | 148.613 ±16.4 | 78.611  ±4 | 539.011  ±41.8 | 0.222  ±0.1 | 0.54  ±0.1 | 0.387  ±0.1 | 35.96  ±60.1 |
| DRN | 177.072  ±9.5 | 99.874  ±6.7 | 559.264  ±41 | 0.155  ±0.1 | 0.56  ±0.1 | 0.401  ±0.1 | 42.143 ±108.9 |
| MR | 152.38  ±29 | 86.163  ±16.5 | 532.305  ±103.8 | 0.264  ±0.1 | 0.533  ±0.2 | 0.414  ±0.1 | 31.245 ±16.1 |
| MRF | 148.109  ±4.3 | 75.995  ±4 | 525.012  ±19.7 | 0.297  ±0.1 | 0.526  ±0.1 | 0.401  ±0.1 | 37.664 ±66.9 |
| PRN | 150.704  ±6 | 86.111  ±3.7 | 590.374  ±33.2 | 0.203  ±0.1 | 0.591  ±0.1 | 0.412  ±0.1 | 30.546 ±10.8 |
| AIHyp | 208.13  ±22.1 | 112.264  ±19 | 415.689  ±28.9 | 0.267  ±0.1 | 0.416  ±0.1 | 0.388  ±0.1 | 34.33  ±43.3 |
| ASHyp | 168.99  ±11.7 | 81.527  ±8.7 | 482.909  ±27 | 0.323  ±0.1 | 0.483  ±0.1 | 0.383  ±0.1 | 70.073 ±267.7 |
| IntHyp | 158.655  ±6.7 | 76.937  ±3.7 | 488.668  ±24.8 | 0.352  ±0.1 | 0.489  ±0.1 | 0.384  ±0.1 | 41.017 ±42.8 |
| Phyp | 204.725 ±26.9 | 111.714 ±17.8 | 526.448  ±43.5 | 0.303  ±0.1 | 0.527  ±0.1 | 0.4  ±0.1 | 49.575 ±136.4 |
| Thalamus | 145.828  ±3.7 | 69.853  ±2.4 | 526.097  ±16.9 | 0.31  ±0.1 | 0.527  ±0.1 | 0.446  ±0.1 | 35.934  ±9 |
| Hippocampus | 168.347  ±4.6 | 79.449  ±2.7 | 418.285  ±13.8 | 0.527  ±0.1 | 0.419  ±0.1 | 0.435  ±0.1 | 35.056  ±15 |
| Amygdala | 170.632  ±4.3 | 80.147  ±2.2 | 464.367  ±12.7 | 0.476  ±0.1 | 0.465  ±0.1 | 0.416  ±0.1 | 43.138  ±88 |

**Table 1:** Means ± SD of each MRI metric in all ROIs in whole group of 96 subjects with complete MRI dataset.
